# Safety and risk factors of TINAVI robot-assisted percutaneous pedicle screw placement in spinal surgery

**DOI:** 10.1186/s13018-022-03271-6

**Published:** 2022-08-08

**Authors:** Ren-Jie Zhang, Lu-Ping Zhou, Lai Zhang, Hua-Qing Zhang, Jian-Xiang Zhang, Cai-Liang Shen

**Affiliations:** Department of Orthopedics and Spine Surgery, The First Affiliated Hospital of Anhui Medical University, 210 Jixi Road, Hefei, 230022 Anhui China

**Keywords:** Robot, Pedicle screw placement, Intra-pedicular accuracy, Proximal facet joint, Risk factor, Spine surgery

## Abstract

**Objective:**

To determine the rates and risk factors of pedicle screw placement accuracy and the proximal facet joint violation (FJV) using TINAVI robot-assisted technique.

**Methods:**

Patients with thoracolumbar fractures or degenerative diseases were retrospectively recruited from June 2018 and June 2020. The pedicle penetration and proximal FJV were compared in different instrumental levels to identify the safe and risk segments during insertion. Moreover, the factors were also assessed using univariate and multivariate analyses.

**Results:**

A total of 72 patients with 332 pedicle screws were included in the current study. The optimal and clinically acceptable screw positions were 85.8% and 93.4%. Of the 332 screws concerning the intra-pedicular accuracy, 285 screws (85.8%) were evaluated as Grade A according to the Gertzbein and Robbins scale, with the remaining 25 (7.6%), 10 (3.0%), 6 (1.8%), and 6 screws (1.8%) as Grades B, C, D, and E. Moreover, in terms of the proximal FJV, 255 screws (76.8%) screws were assessed as Grade 0 according to the Babu scale, with the remaining 34 (10.3%), 22 (6.6%), and 21 screws (6.3%) as Grades 1, 2, and 3. Furthermore, the univariate analysis showed significantly higher rate of penetration for patients with age < 61 years old, sex of female, thoracolumbar insertion, shorter distance from skin to insertion point, and smaller facet angle. Meanwhile, the patients with the sex of female, BMI < 25.9, grade I spondylolisthesis, lumbosacral insertion, longer distance from skin to insertion point, and larger facet angle had a significantly higher rate of proximal FJV. The outcomes of multivariate analyses showed that sex of male (adjusted OR 0.320, 95% CI 0.140–0.732; *p* = 0.007), facet angle ≥ 45° (adjusted OR 0.266, 95% CI 0.090–0.786; *p* = 0.017), distance from skin to insertion point ≥ 4.5 cm (adjusted OR 0.342, 95% CI 0.134–0.868; *p* = 0.024), and lumbosacral instrumentation (adjusted OR 0.227, 95% CI 0.091–0.566; *p* = 0.001) were independently associated with intra-pedicular accuracy; the L5 insertion (adjusted OR 2.020, 95% CI 1.084–3.766; *p* = 0.027) and facet angle ≥ 45° (adjusted OR 1.839, 95% CI 1.026–3.298; *p* = 0.041) were independently associated with the proximal FJV.

**Conclusion:**

TINAVI robot-assisted technique was associated with a high rate of pedicle screw placement and a low rate of proximal FJV. This new technique showed a safe and precise performance for pedicle screw placement in spinal surgery. Facet angle ≥ 45° is independently associated with both the intra-pedicular accuracy and proximal FJV.

**Supplementary Information:**

The online version contains supplementary material available at 10.1186/s13018-022-03271-6.

## Introduction

The pedicle screw placement has been widely applied in the treatment of spinal fractures, tumor, infections, deformities, and degenerative diseases for the improvement of stability after the spinal reconstruction. However, due to the spinal cord, nerve root, and blood vessels adjacent to the pedicle, the malposition of screws might contribute to severe complications, the rate of which was reported as 4.2% of patients [1]. Besides, the proximal facet joint violation (FJV), one of the most common complications of malposition, has been regarded as a crucial risk factor for adjacent segment degeneration (ASD) after surgery [2, 3]. Moreover, Levin et al. [4] reported that proximal FJV was independently associated with a higher reoperation rate and diminished improvement in quality of life.

Initially, the pedicle screws were placed in open methods with inevitable need for paravertebral muscle dissection resulting in excessive bleeding and trauma of soft tissues. With the wide acceptance of minimally invasive concepts, various insertion techniques, such as the percutaneous fluoroscopy-guided, patient-specific template-guided, the computer-assisted, and robot-assisted (RA) navigation techniques, have been designed to guide the pedicle screw placement for the enhancement of insertion accuracy, protection of proximal facet joint, and reduction of severe complication including neurological, vascular, and muscular injuries [5, 6]. Among them, the widely used robots of SpineAssist, Renaissance, and Mazor X (Mazor Robotics Ltd., Caesarea, Israel) were reported to enable less damage to soft tissues, higher intra-pedicular accuracy of 85.0–98.2% [7–13], and lower rates of proximal FJV of 0–5% [9, 13, 14] with intraoperative navigation, automatic trajectory determination, and reduced manual errors [15]. With the clinical application of orthopedic robots, spine surgery has entered the era of intelligence.

Recent years, a new robot system named TiRobot (TINAVI Medical Technologies Co. Ltd.) has been introduced in orthopedic surgery, which received China Food and Drug Administration approval in 2016 [16]. A series of studies conducted by Tian et al. [16–19] reported that the TiRobot system led to higher intra-pedicular accuracy rates of 93.2–98.2% [16–18] and lower proximal FJV rates of 0–13.0% [16, 18, 19] in a single trial center. However, the accuracy of RA pedicle screw placement was unstable and controversial. Meanwhile, the studies regarding the intra-pedicular and proximal facet joint accuracy of TiRobot system are still rare, and the risk factors of the pedicle screw placement accuracy and proximal FJV with TiRobot system need further identification. Thus, this study was performed to evaluate the radiographic outcomes of intra-pedicular accuracy and proximal FJV in TINAVI RA technique and analyze the risk factors affecting the inaccuracy of pedicle screw placement and proximal FJV to provide references for surgeons.

## Method

### Study design and patient selection

A consecutive series of patients who underwent TINAVI RA percutaneous pedicle screw placement for the treatment of thoracolumbar fractures or the degenerative diseases between June 2018 and June 2020 were, respectively, recruited, and all the operations were conducted by a surgical team. The study was approved by hospital's institutional review board with all the included patients signing informed consent forms.

The inclusion criteria were as follows: (1) patients older than 18 years old; (2) patients diagnosed of thoracolumbar fractures or degenerative diseases including lumbar disk herniation, spinal stenosis, grade I spondylolisthesis, and mild degenerative scoliosis; (3) patients undergoing TINAVI RA percutaneous insertion, regardless of purely instrumental fixation or decompression along with intervertebral fusion; (4) insertion segments ranging from T11 to S1; and (5) patient receiving postoperative three-dimensional (3D) CT examination. The exclusion criteria consisted of the following: (1) patients with a history of previous surgery on the thoracolumbar segments, and revision surgery in the same segments; (2) patients with destruction of the vertebral body caused by tuberculosis infection or tumor, and unclear anatomy of the surgical segments; and (3) patients with active local or systematic infection, spinal tumor, or the tuberculosis.

Patients information on age, gender, body mass index (BMI), and diagnosis before surgery was obtained from medical records with the consent from patients.

### Robot-assisted percutaneous insertion technique

The percutaneous pedicle screw placements were performed with the TiRobot system composed of three basic elements: an optical tracking device, a workstation for preoperative planning and intraoperative controlling, and a robotic arm [16].

After general anesthesia with endotracheal intubation, the patient took the prone position. Then, the position plate was located and the projection of the planned vertical body pedicle on skin was marked. The pedicle screws were inserted in according with the original method under the guidance of TiRobot system [16].

As for the patients suffering from fractures, the pedicle screw was inserted directly along the guide wire, and the incision was closed after the fracture rehabilitation performed with the screw rod. In contrast, regarding the patients suffering from the degenerative diseases, the screw is placed on the non-decompression side after the insertion of guide wire under the robotic guidance, whereas the trajectory of screw is prepared along the guide wire on the decompression side, and then, the screw is placed after transforaminal lumbar interbody fusion (TLIF) [20].

### Outcome measures

**Fig. 1:**
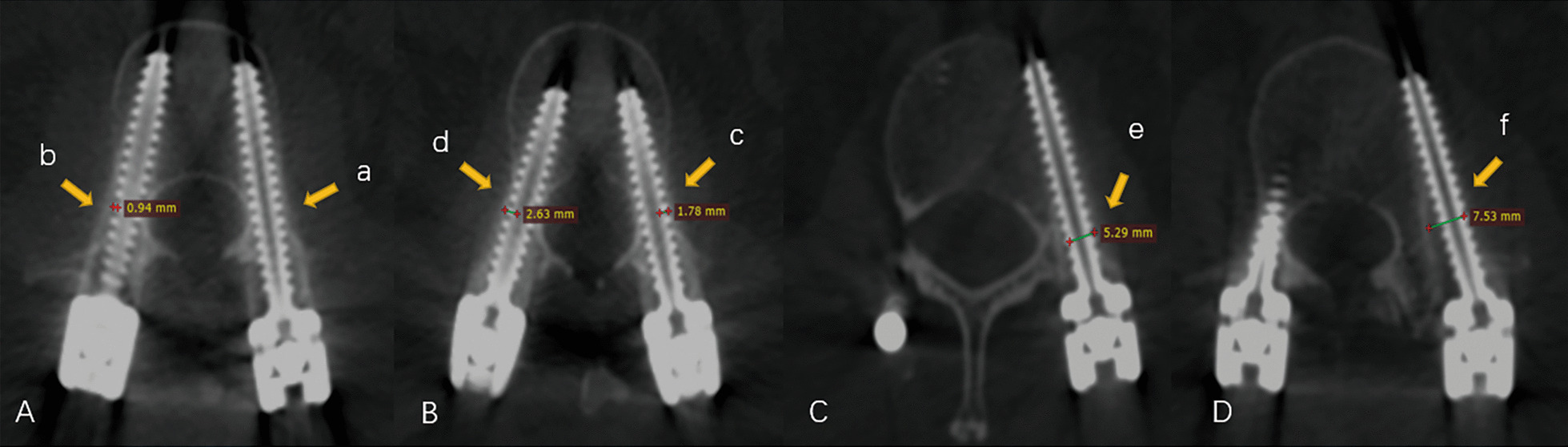
Intra-pedicular accuracy according to the Gertzbein–Robbins scale in robot-assisted technique. A. Grade A, screw position completely within the pedicle (**a**); Grade B, cortical breach of 0.94 mm (**b**); B. Grade B, cortical breach of 1.78 mm (**c**); Grade C, cortical breach of 2.63 mm (**d**); C. Grade D, cortical breach of 5.29 mm (**e**); and D. Grade E, cortical breach of 7.53 mm (**f**)

**Fig. 2:**
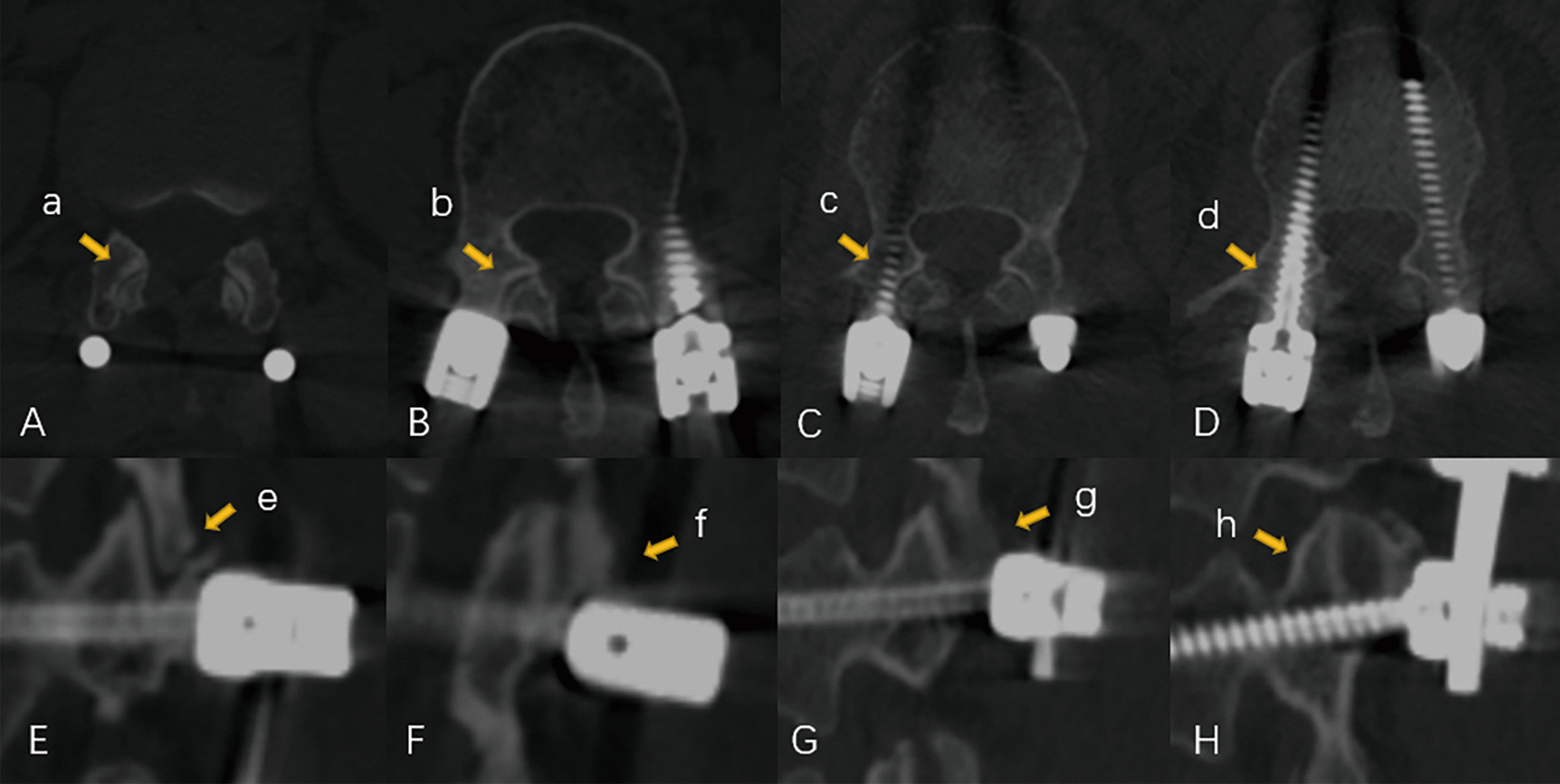
Proximal facet joint violation in accordance with the Babu scale in robot-assisted technique. A. Grade 0, screw not in facet, transverse position (**a**); B. Grade 1, screw in lateral facet but not in facet articulation, transverse position (**b**); C. Grade 2, penetrating facet articulation less than 1 mm, transverse position (**c**); D. Grade 3, traveling within facet articulation, transverse position (**d**); E. Grade 0, screw not in facet, sagittal position (**e**); F. Grade 1, screw in lateral facet but not in facet articulation, sagittal position (**f**). G. Grade 2, penetrating facet articulation less than 1 mm, sagittal position (**g**); and H. Grade 3, traveling within facet articulation, sagittal position (**h**)

The accuracy of pedicle screw placement was assessed in accordance with the Gertzbein and Robbins scale [21] with the following grades: A, the screw completely contained in the pedicle; B, the cortical penetration of less than 2 mm; C, the cortical penetration of 2 mm or more but less than 4 mm; D, the cortical penetration of 4 mm or more but less than 6 mm; and E, the cortical penetration of 6 mm or more. (Fig. 1) Grade A was regarded as optimal screw position, and Grades A and B indicated clinically acceptable screw position, whereas Grades C, D, and E were considered as misplacement. Besides, the evaluation of proximal FJV was according to the Babu scale [22] with the following grades: 0, screw not in facet; (1), screw in lateral facet but not in facet articulation; (2), penetration of facet articulation less than 1 mm; and (3), penetration of facet articulation more than 1 mm or traveling within facet articulation. (Fig. 2) Moreover, the pedicle penetration and proximal FJV were also compared in different instrumental levels to identify the safe and risk segments during insertion.

The following factors that possibly affected the accuracy of the pedicle screw placement and proximal FJV were also collected during the assessment of the postoperative CT scan: proximal facet joint degeneration, facet angle, axial pedicle angle, sagittal pedicle angle, distance from skin to insertion point. The proximal facet joint degeneration was measured according to the Pathria classification [23]. The radiographic data were independently evaluated by a radiologist and a surgeon on the postoperative CT scans with a 1-mm thickness slice. If the two evaluators were inconsistent on the results, the final decision was made by the third surgeon.

### Statistical analysis

The statistical analyses were performed with SPSS version 23.0. The continuous variables were described as the mean and standard deviation and the categorical variables as absolute (no.) and relative (%) frequencies. For statistical purpose, the rates and different grades of the accuracy of pedicle insertion and violation of proximal facet joint were analyzed in each instrumented level. Moreover, possible factors associated with intra-pedicular and proximal facet joint accuracy were gender, age, BMI, side of screw, diagnosis of grade I spondylolisthesis and mild degenerative scoliosis, facet degeneration, instrumented levels (thoracolumbar or lumbosacral instruments), depth of surgical field (distance of entry point from skin), facet angle, axial pedicle angle, and sagittal angle. After the univariate analyses of the above factors, the remaining issues with a significant level < 0.05 in univariate analyses were summarized to the final logistics regression modal of multivariate analyses. As for the intra-pedicular accuracy, the variables included in the multivariate analyses were gender, age, instrumented levels, depth of surgical field, and facet angle, while for the proximal FJV, the variables were gender, BMI, diagnosis of grade I spondylolisthesis, instrumented levels, depth of surgical field, and facet angle. The level of significance was defined as alpha = 0.05.

### Result

A total of 72 patients with 332 pedicle screws were included in the study. The demographic characteristics and screws-related data are summarized in Tables 1 and 2, respectively. The overall patient mean age of the 32 females and 40 males was 50.17 ± 10.93 years old (range 28–72), and the mean value of the BMI was 23.54 ± 3.98 (range 18.3–36.7). The diagnoses were thoracolumbar fracture in 34 cases, lumbar disk hernia in 24 cases, lumbar canal stenosis in 2 cases, grade I spondylolisthesis in 7 cases, and mild degenerative scoliosis in 5 cases. Moreover, of the 322 screws insertions, the most frequently inserted level was L5 (70 screws) followed by L4 (60 screws), L1 (54 screws), and T12 (44 screws). All the screws were inserted in bilateral sides. Meanwhile, 188 proximal facet joints (56.6%) were regarded as mild osteoarthritis. The mean values of axial and sagittal pedicle angles were 14.02 ± 4.92° and 2.98 ± 6.63°, respectively. Furthermore, the mean value distance from skin to insertion point was 5.35 ± 0.90 cm.

**Table 1:** Demographics and anatomical characteristics

Variables	*N* = 72
Mean age (year)	50.17 ± 10.93
*Gender*	
Male	32 (44.4%)
Female	40 (55.6%)
Body mass index (m/kg^2^)	23.54 ± 3.98
*Diagnosis*	
Fracture	34 (47.2%)
Lumbar disk hernia	24 (33.3%)
Lumbar canal stenosis	2 (2.8%)
Spondylolisthesis (grade I)	7 (9.7%)
Mild degenerative scoliosis	5 (6.9%)

**Table 2:** Screw-related variables

Variables	*N* = 332
*Instructed level*	
T11	16 (4.8%)
T12	44 (13.3%)
L1	54 (16.3%)
L2	44 (13.3%)
L3	20 (6.0%)
L4	60 (18.1%)
L5	70 (21.1%)
S1	24 (7.2%)
*The side of screw*	
Right	166 (50%)
Left	166 (50%)
*Facet joint osteoarthritis*	
Normal	45 (13.6%)
Mild	188 (56.6%)
Moderate	79 (23.8%)
Severe	20 (6.0%)
Axial pedicle angle (°)	14.02 ± 4.92
Sagittal pedicle angle	2.98 ± 6.63
Distance from skin to insertion point (cm)	5.35 ± 0.90

Of the 332 screws concerning the pedicle screw accuracy, 285 screws (85.8%) were evaluated as Grade A according to the Gertzbein and Robbins scale, with the remaining 25 (7.6%), 10 (3.0%), 6 (1.8%), and 6 screws (1.8%) as Grades B, C, D, and E, respectively. Optimal and clinically acceptable screw positions were achieved in 85.8% and 93.4% of all pedicle screws, respectively. The cortical encroachments in pedicles were found for 47 screws. Among the penetrations, the lateral cortex was the most frequently breached (74.5%), followed by the medial (21.2%) and inferior (4.3%) cortexes. Meanwhile, the risk of pedicle penetration at L5 level was significantly lower than that at other segments (Raw OR 0.222, 95% CI 0.067–0.737; *p* = 0.013).

In terms of the proximal FJV, 255 screws (76.8%) screws were assessed as Grade 0 according to the Babu scale, with the remaining 34 (10.3%), 22 (6.6%), and 21 screws (6.3%) as Grades 1, 2, and 3. The risk of proximal FJV in T12 insertion was 3.390 times than other levels (*p* = 0.029), whereas L5 insertion was associated with significantly higher risk of 3.153 times than others (*p* < 0.001). Besides, the screw head was the most frequent region responsible for proximal FJV (80.5%), followed by screw shaft (19.5%). There is no violation from the rod. The rates of intra-pedicular and proximal facet joint accuracy and the related raw ORs in different instrumented levels are shown in Tables 3 and 4.

**Table 3:** Instrumented levels and risks for intra-pedicular accuracy

Level	A	B	A + B	C	D	E	Intact (%)	Breached (%)	Raw OR (95%Cl)	*p*
T11	12	1	13	1	0	2	12 (3.6)	4 (1.2)	2.166 (0.653–6.862)	0.364
T12	33	5	38	2	2	2	33 (9.9)	11 (3.3)	2.333 (1.084–5.022)	0.270
L1	42	3	45	6	2	1	42 (12.7)	12 (3.6)	1.984 (0.953–4.128)	0.063
L2	34	7	41	0	2	1	34 (10.2)	10 (3.0)	1.995 (0.910–4.374)	0.080
L3	17	3	20	0	0	0	17 (5.1)	3 (0.9)	1.075 (0.302–3.820)	0.911
L4	56	4	60	0	0	0	56 (16.9)	4 (1.2)	0.380 (0.131–1.104)	0.066
L5	67	2	69	1	0	0	67 (20.2)	3 (0.9)	0.222 (0.067–0.737)	0.013
S1	24	0	24	0	0	0	24 (7.2)	0 (0.0)	0	0.998
Total	285	25	310	10	6	6	285 (85.8)	47 (14.2)	–	–

*A* optimal position, *A* + *B* clinically acceptable position

**Table 4:** Instrumented levels and risks for proximal facet joint violation

Level	0	1	2	3	Intact (%)	Violated (%)	Raw OR (95%Cl)	*p*
T11	14	1	0	1	14 (4.2)	2 (0.6)	0.459 (0.102–2.066)	0.462
T12	40	3	0	1	40 (12.0)	4 (1.2)	0.295 (0.102–0.851)	0.029
L1	45	5	3	1	45 (13.6)	9 (2.7)	0.618 (0.287–1.329)	0.214
L2	36	4	2	2	36 (10.8)	8 (2.4)	0.705 (0.313–1.589)	0.398
L3	15	3	0	2	15 (4.5)	5 (1.5)	1.111 (0.390–3.162)	0.843
L4	42	8	4	6	42 (12.7)	18 (5.4)	1.547 (0.830–2.885)	0.168
L5	41	10	11	8	41 (12.3)	29 (8.7)	3.153 (1.785–5.572)	< 0.001
S1	22	0	2	0	22 (6.6)	2 (0.6)	0.282 (0.065–1.229)	0.124
Total	255	34	22	21	255 (76.8)	77 (23.2)	–	–

The summary and characteristics of adverse events are displayed in Additional file 1. Regarding the complications, no patients underwent intraoperative revision due to screw malposition or postoperative revision due to screw malposition, and no neurological symptoms resulted from screw insertion. Furthermore, one patient suffered from wound infection after surgery with no need for another debridement surgery.

Factors that affected the intra-pedicular and proximal facet joint accuracy of robotic guidance percutaneous approach were explored with univariate analyses (Additional file 2). Regarding the intra-pedicular accuracy, the patients with the age < 61 years old, sex of female, thoracolumbar insertion, shorter distance from skin to insertion point, and smaller facet angle had a significantly higher rate of penetration. As for the proximal FJV, the patients with the sex of female, BMI < 25.9, grade I spondylolisthesis, lumbosacral insertion, longer distance from skin to insertion point, and larger facet angle had a significantly higher rate.

After the control of confounders, the multivariate analyses of associated with the intra-pedicular and proximal facet joint accuracy were performed concerning the issues in univariate analyses with a significant level < 0.05 (Table 5). The result showed that the sex of male (adjusted OR 0.320, 95% CI 0.140–0.732; *p* = 0.007), facet angle ≥ 45° (adjusted OR 0.266, 95% CI 0.090–0.786; *p* = 0.017), distance from skin to insertion point ≥ 4.5 cm (adjusted OR 0.342, 95% CI 0.134–0.868; *p* = 0.024), and lumbosacral instrumentation (adjusted OR 0.227, 95% CI 0.091–0.566; *p* = 0.001) were independently associated with intra-pedicular accuracy. Meanwhile, the L5 insertion (adjusted OR 2.020, 95% CI 1.084–3.766; *p* = 0.027) and facet angle ≥ 45° (adjusted OR 1.839, 95% CI 1.026–3.298; *p* = 0.041) were independently associated with proximal FJV.

**Table 5:** Logistic regression model of variables associated with intra-pedicular and proximal facet joint accuracy, respectively

Variables group	Raw OR (95% Cl)	Adjusted OR (95% Cl)	*p*
*Pedicle cortex penetration*			
Sex of male	0.494 (0.263–0.925)	0.320 (0.140–0.732)	0.007
Facet angle ≥ 45°	0.241 (0.091–0.642)	0.266 (0.090–0.786)	0.017
Depth of surgical field ≥ 4.5 cm	0.173 (0.088–0.340)	0.342 (0.134–0.868)	0.024
Lumbosacral instrumentation	0.199 (0.095–0.417)	0.227 (0.091–0.566)	0.001
*Facet joint violation*			
L5 insertion	3.153 (1.785–5.572)	2.020 (1.084–3.766)	0.027
Facet angle ≥ 45°	2.197 (1.268–3.807)	1.839 (1.026–3.298)	0.041

## Discussion

The accuracy of pedicle screw insertion is especially crucial because of the severe consequences followed by malposition including neurological and vascular injuries, revision surgery, and spinal instability [13, 16]. Meanwhile, the proximal FJV has been regarded as an independent risk factor for ASD, finally resulting in higher reoperation rate and diminished improvement in quality of life [3, 4]. Thus, the safety of pedicle screw placement has increasingly drawn attention from spinal surgeons. With the application of robotic guidance systems, higher rate of intra-pedicular accuracy and lower rate of proximal FJV in RA technique than those in percutaneous fluoroscopy-guided and freehand techniques have been reported in recent years [5, 7–9, 13, 14, 16–19]. However, the rates of intra-pedicular accuracy and proximal FJV were quite different in previously published studies, and compared with the Mazor RA techniques, the studies on TINAVI systems are rare. Furthermore, the risk factors that affected the outcomes of pedicle screw placement remain controversial and no consensus has been reached in clinical research. Here, we evaluated the safety of pedicle screw placement with TINAVI RA techniques in terms of the intra-pedicular accuracy and proximal FJV and analyzed the risk factors associated with the two issues in screw implantation.

Many studies have concluded that RA technique showed improvement of intra-pedicular accuracy at the rates of 86.16–98.2% than conventional techniques [7, 8, 16–18, 24]. However, the previously published studies also showed that the RA technique performed no clear advantage in terms of intra-pedicular accuracy rates of 78.8–93.7% over the conventional techniques [5, 9, 13, 25]. Hyun et al. [9] conducted a RCT of Mazor robotic-guided versus freehand surgery and found that all screws were optimal positions in RA group and 98.6% screws were graded as optimal positions, showing no remarkable difference. Kim et al. [13] also concluded that the intra-pedicular rate of 93.7% in the Mazor robotic-guided group was significantly similar to that of 91.9% in the freehand group. In the current study, we found that the optimal (Grade A) and clinically acceptable (Grade A + B) screw position was 85.8% and 93.4%, respectively, indicating promising results. Moreover, the main direction of screw deviations in pedicle was lateral, followed by medial and inferior breaches. The results might be explained as follows. First, based on the intraoperative 3D images where the anatomical structures, especially the abnormal and degenerative parts, were clearly displayed, the RA system could automatically identify the optimal entry point and trajectory direction for the safety of the pedicle cortex. Then, the implantation procedure was automatically performed according to the preplanned satisfactory trajectory in order to ensure the intra-pedicular accuracy. Second, the robot can repeat complex tasks precisely, thereby avoiding the limitation of human manual errors. Third, RA technique can minimize the resistance from the soft tissues around the spine compared to freehand method. After the guide wire was inserted under the guidance of a robotic arm, the screws were placed through the guiding tube into the pedicle. In contrast, the selection of entry point and the adjustment of trajectory would be biased due to the pulling and blocking of the paravertebral muscles in freehand surgery, leading to higher risk of screw malposition. Fourth, the RA insertion has a short learning curve because of the automatic procedures and the precision of implantation. Previous studies reported that the surgical outcomes from the initial experience to practiced experience showed no remarkable difference [26, 27]. Thus, surgical skills are relatively less demanding, thereby indicating that RA technique may prevent malposition by reducing unnecessary manual mistakes. Besides, the screw deviations directed medially or inferiorly were more dangerous than lateral deviation due to the higher risk of the relevant severe complications, such as spinal cord and nerve root injuries. Thus, when preplanning the trajectory of insertion, the surgeons mainly focus on the avoidance of the penetration of medial and inferior cortex. Consequently, the rate of lateral deviations relatively increased with the rates of medial and inferior penetrations greatly reduced, especially for the thoracic vertebra with thinner pedicles.

The importance of proximal facet joint protection has been increasingly realized in recent years, and the violation might lead to the spinal instability in adjacent segments and reduce the load-bearing ability, eventually resulting in the radiographic ASD [3, 28]. The studies regarding the superiority of RA versus conventional freehand techniques remained controversial. On the one hand, some studies revealed that the RA pedicle screw placement was associated with significantly fewer FJV (0.00–2.84%) than the conventional trajectory freehand implantation (15.85–100.00%) [2, 13, 14, 16, 18, 29–32]. On the other hand, in a prospective RCT conducted by Hyun et al. [9], no remarkably statistical difference was noted regarding the rates of proximal FJV between the RA and freehand techniques (0.00% vs. 0.71%). In a similar study, Archavlis et al. [14] also reported that the rate of proximal FJV in the RA group was similar to that in the freehand group (5% vs. 6%). In the present study, we found that the proximal FJV was 23.2%, which was lower than that in freehand method but relatively higher than that in RA method from most of the previous published studies. The results might be interpreted as that majority of pedicle screws were inserted in thoracolumbar levels with the patients diagnosed of vertebral fractures. However, we concluded in the current study that the FJVs in the thoracolumbar levels were remarkably higher than those in the lumbosacral levels due to the thinner diameter of pedicle in the thoracolumbar segments. Thus, the overall rate of FJV may be affected by the higher rate of thoracolumbar FJV. Furthermore, the reduction of proximal FJV might result from the precision of pedicle screw placement. With the 3D images, the anatomical landmarks on the vertebra with hypoplastic or degenerative diseases can be displayed clearly with RA method. The RA system can identify the optimal entry point and trajectory and insert screws more accurately for the safety of facet joint by enlarging the distance between the facet and screws. Moreover, the advantages of muscle resistance and the short learning curve via RA method are also beneficial to facet protection.

The factors that affected the intra-pedicular accuracy and proximal FJV were also explored with univariate and multivariate analyses. As for the pedicle screw placement accuracy, our multivariate regression analysis demonstrated that lumbosacral instrumentation was 0.227 times likely to breach the cortex because of a larger diameter of pedicle in the lower lumbar segments. Thus, the occurrence of screw deviation causing severe penetration in lumbosacral segments might decrease than that in thoracolumbar segments. Moreover, we also found that the facet angle ≥ 45° and the depth of surgical field ≥ 4.5 cm are the protective factors in pedicle screw placement, which might result from the fact that in the lumbosacral levels, the facet angle was larger and the mean distance from the entry point to the skin was further than those in the thoracolumbar levels. Because of the higher rate of accuracy in lumbosacral instrumentation, the distance from entry point to screw ≥ 4.5 cm was less likely to breach the cortex. Moreover, we also found that the male patients suffered from less screw penetration.

In terms of the independent factors that affected the cranial FJV, our multivariate analysis showed that the L5 insertion was 2.02 times more likely to breach the facet. Tian et al. [33] identified an increasing trend of proximal FJV rates ranging from 1.5%, 7.4%, 17.6%, 30.9% to 42.6% with the levels of vertebrae dropping from L1 to L5. Besides, Moshirfar et al. [34] inserted the screws with freehand method, and it was found that the proximal FJV in the L5 level (48%) was significantly higher than that of other levels. In L5 segment, the ideal entry point was deviated to the outside with the increment of valgus angle. Meanwhile, the articular surface of the facet joint also shifted to the lateral and coronal planes, blocking the insertion trajectory and ultimately increasing proximal FJV [22, 30]. Moreover, patients with facet angle ≥ 45° were 1.89 times more likely have a proximal FJV than patients with facet angle < 45°. When the facet angle increases, the surface of facet joint gradually deviates to the coronal position, thereby blocking the trajectory of screws [28]. Another explanation might be that the facet angle in the lower lumbar is larger than that in the upper lumbar, and the lumbar lordosis is mainly manifested in the lower lumbar. Thus, there is more overlap between the facet joints and the pedicle direction, which also blocks the pathway of screws [19]. Whether by freehand technique or by robot-assisted technique, the larger facet angle is an independent factor increasing the proximal FJV based on our previous investigation and the current study should be valued [35]. The preoperative measurement of facet angle is recommended.

There are some potential limitations in the current study. First, no control group was included in the study and only the data of RA technique were reported without comparative results. Although we compared the outcomes with those from previously published studies, the higher biases appeared. Second, the overall intra-pedicular accuracy and proximal FJV consisted of thoracic and lumbar segments. The results may be affected due to the different anatomical characteristics. Third, because this study was focused on the RA system, the results were only applicable to the RA insertion instead of the universal operation protocol. Fourth, we only reported the radiographic results in a short-term follow-up period. Nevertheless, the postoperative short-term outcomes are crucial for a novel technique regarding the accuracy of pedicle screw placement. Continued assessment of safety and accuracy of screw placement in long-term follow-up using TINAVI RA technique is needed in the future work.

## Conclusion

TINAVI robot-assisted technique was associated with a high rate of pedicle screw placement and a low rate of proximal FJV. This technique showed a safe and precise performance for pedicle screw placement in spinal surgery.

## Supplementary Information

**Additional file 1**: Summary and characteristics of adverse events.

**Additional file 2**: Univariate analyses of factors associated with intra-pedicular and proximal facet joint accuracy, respectively.

## Data Availability

All data generated or analyzed during this study are included in this published article and its supplementary information files.

## Abbreviations

ASD: Adjacent segment degeneration
FJV: Proximal facet joint violation
RA: Robot-assisted
BMI: Body mass index
TLIF: Transforaminal lumbar interbody fusion
