# Supplementary material for: Safety and risk factors of TINAVI robot-assisted percutaneous pedicle screw placement in spinal surgery
Source: J Orthop Surg Res. 2022 Aug 8;17:379. doi: 10.1186/s13018-022-03271-6 (PMC9361479; doi:10.1186/s13018-022-03271-6)
Supplement: Supplementary file 1 — Additional file 1: Summary and characteristics of adverse events. [file 13018_2022_3271_MOESM1_ESM.docx]

**Supplemental File 1 Summary and characteristics of adverse events**

| **Adverse events** | **Number (percentage)** |
| --- | --- |
| ***Deviation of screw in pedicle*** |  |
| Superior | 0 (0) |
| Inferior | 2 (4.3%) |
| Medial | 10 (21.2%) |
| Lateral | 35 (74.5%) |
| Penetration | 47 (100%) |
| ***Screw region for FJV*** |  |
| Screw shaft | 15 (19.5%) |
| Screw head | 62 (80.5%) |
| Rod | 0 (0) |
| Violation | 77 (100%) |
| ***Complications*** |  |
| Intraoperative revision due to screw malposition | 0 (0) |
| Postoperative revision due to screw malposition | 0 (0) |
| Neurological symptoms due to screw insertion | 0 (0) |
| Wound infections | 1 (1.4%) |
| Patients | 72 (100%) |
