# Supplementary material for: Safety and risk factors of TINAVI robot-assisted percutaneous pedicle screw placement in spinal surgery
Source: J Orthop Surg Res. 2022 Aug 8;17:379. doi: 10.1186/s13018-022-03271-6 (PMC9361479; doi:10.1186/s13018-022-03271-6)
Supplement: Supplementary file 2 — Additional file 2: Univariate analyses of factors associated with intra-pedicular and proximal facet joint accuracy, respectively. [file 13018_2022_3271_MOESM2_ESM.docx]

**Supplemental File 2 Univariate analyses of factors associated with intra-pedicular and proximal facet joint accuracy, respectively**

| Variable | No. of screws | Pedicle | |  |  | Facet joint | |  |  |
| --- | --- | --- | --- | --- | --- | --- | --- | --- | --- |
|  |  | Intra-pedicular screw | Extra-pedicular screw | t/Z/χ2 | P  Value | Intra-articular screw | Extra- articular screw | t/Z/χ2 | p |
| Sex | 332 |  |  | 4.984 | 0.026 |  |  | 4.031 | 0.045 |
| Female |  | 120 | 28 |  |  | 106 | 42 |  |  |
| Male |  | 165 | 19 |  |  | 149 | 35 |  |  |
| Age (years) | 332 |  |  | 3.697 | 0.041 |  |  | 0.103 | 0.853 |
| ＜61 |  | 239 | 45 |  |  | 219 | 65 |  |  |
| ≥ 61 |  | 46 | 2 |  |  | 36 | 12 |  |  |
| Body mass index (kg/m2) | 332 |  |  | 2.253 | 0.133 |  |  | 4.729 | 0.030 |
| <25.9 |  | 214 | 40 |  |  | 188 | 66 |  |  |
| ≥25.9 |  | 71 | 7 |  |  | 67 | 11 |  |  |
| Side of screw | 332 |  |  | 2.008 | 0.157 |  |  | 0.829 | 0.435 |
| Right |  | 138 | 28 |  |  | 124 | 42 |  |  |
| Left |  | 147 | 19 |  |  | 131 | 35 |  |  |
| Spondylolisthesis | 332 |  |  | 1.522 | 0.217 |  |  | 5.230 | 0.022 |
| Yes |  | 28 | 2 |  |  | 18 | 12 |  |  |
| No |  | 257 | 45 |  |  | 237 | 65 |  |  |
| Scoliosis | 332 |  |  | 0.776 | 0.378 |  |  | 0.554 | 0.457 |
| Yes |  | 19 | 1 |  |  | 14 | 6 |  |  |
| No |  | 266 | 46 |  |  | 241 | 71 |  |  |
| Facet degeneration | 332 |  |  | 0.390 | 0.361 |  |  |  |  |
| Yes |  | 244 | 43 |  |  | 221 | 66 | 0.046 | 0.850 |
| No |  | 41 | 4 |  |  | 34 | 11 |  |  |
| Instrumented levels | 332 |  |  | 21.277 | ＜0.001 |  |  | 12.621 | ＜0.001 |
| Thoracolumbar (T11-L2) |  | 121 | 37 |  |  | 135 | 23 |  |  |
| Lumbosacral (L3-S1) |  | 164 | 10 |  |  | 120 | 54 |  |  |
| Depth of surgical field (cm) | 332 | 5.43 ± 0.86 | 4.84 ± 0.97 | -4.410 | ＜0.001 | 5.30 ± 0.90 | 5.53 ± 0.89 | -1.981 | 0.048 |
| Facet angle (°) | 278 | 42.91 ± 13.02 | 35.13 ± 9.56 | -3.364 | 0.001 | 40.89 ± 12.96 | 44.85 ± 12.27 | -2.445 | 0.014 |
| Axial pedicle angle (°) | 332 | 14.03 ± 4.97 | 13.96 ± 4.69 | 0.100 | 0.921 | 14.21 ± 4.93 | 13.41 ± 4.89 | 1.251 | 0.212 |
| Sagittal pedicle angle (°) | 332 | 2.85 ± 6.55 | 3.81 ± 7.09 | -0.920 | 0.358 | 2.85 ± 6.51 | 3.44 ± 7.01 | -0.692 | 0.489 |
